# Bis-Thiourea Quaternary Ammonium Salts as Potential Agents against Bacterial Strains from Food and Environmental Matrices

**DOI:** 10.3390/antibiotics10121466

**Published:** 2021-11-28

**Authors:** Maria Grazia Bonomo, Teresa Giura, Giovanni Salzano, Pasquale Longo, Annaluisa Mariconda, Alessia Catalano, Domenico Iacopetta, Jessica Ceramella, Maria Stefania Sinicropi, Carmela Saturnino

**Affiliations:** 1 Department of Sciences, University of Basilicata, Viale dell’Ateneo Lucano 10, 85100 Potenza, Italy; mariagrazia.bonomo@unibas.it (M.G.B.); teresa_albano@hotmail.it (T.G.); giovanni.salzano@unibas.it (G.S.); annaluisa.mariconda@unibas.it (A.M.); carmela.saturnino@unibas.it (C.S.); 2 Spinoff TNcKILLERS, Viale dell’Ateneo Lucano 10, 85100 Potenza, Italy; 3 Department of Chemistry and Biology, University of Salerno, Via Giovanni Paolo II 132, 84084 Fisciano, Italy; plongo@unisa.it; 4 Department of Pharmacy-Drug Sciences, University of Bari “Aldo Moro”, Via Orabona 4, 70126 Bari, Italy; 5 Department of Pharmacy, Health and Nutritional Sciences, University of Calabria, 87036 Arcavacata di Rende, Italy; domenico.iacopetta@unical.it (D.I.); jessica.ceramella@unical.it (J.C.); s.sinicropi@unical.it (M.S.S.)

**Keywords:** antibacterial activity, thiourea derivatives, bacterial strains, food and environmental matrices

## Abstract

In recent years, the phenomenon of antibiotic resistance in hospitals, communities and the environment has increasingly grown, so antibiotic resistance has become an urgent problem that requires a decisive and global intervention. Incorrect/unnecessary use of antibiotics contributes to increase the ability of microorganisms to develop resistance faster and faster. Research efforts must, therefore, be made to ensure a future in which antibiotic drugs will still be useful in combating infectious diseases. The search for new antibacterial compounds is fundamental. In this study, the antimicrobial activity of the compounds was evaluated against selected bacterial strains from food and environmental matrices by using the Agar Well Diffusion Assay. A total of thirty-six Gram-positive and Gram-negative bacteria were employed to determine the action spectrum and the antimicrobial effectiveness of a small series of thiourea derivatives. Results showed that the highest activities were found for compounds **1** and **4**. The important role of the alkyl chain length and/or guanidine moiety in the width of action spectrum was evidenced. Further studies will allow evaluating the efficacy of the inhibiting action and the molecular mechanisms underlying this activity in order to identify compounds capable of counteracting the phenomenon of antibiotic resistance and to identify possible future applications of these newly synthesized compounds that have shown a high bactericidal action potential.

## 1. Introduction

According to a recent European study, Italy ranks first in Europe for the number of infections (more than 200,000) and deaths (about 10,000) caused by antimicrobial resistance (AMR). The continuous emergence of AMR restricts efficacy in treating infectious diseases [1]. Both Italian and International Epidemiological data indicate that AMR by 2050 cause 10 million deaths, due to bacterial infections from multi-resistant bacteria [2,3], representing one of the main threats for health systems around the world [4]. Following the recommendations of international institutions, in Italy, in 2017 the “National Antimicrobial Resistance Plan (PNCAR) 2017–2020” was approved [5]. It constitutes a guideline document for the fight against antibiotic resistance at the national, regional and local levels and represents an opportunity for better coordination and greater incisiveness of the actions in order to obtain significant improvements. One of the main objectives of the strategy is the reduction of the frequency of infections with resistant bacteria, also related to hospital health care. Specifically, objectives are measured by three indicators, which each region undertakes to implement through specific plans, such as monitoring the consumption of antibiotics in hospitals and territories, enhancing awareness in the community of the appropriate use of antibiotics and identifying surveillance procedures and controls for health care-related infections. Since 2001, in Italy, the Istituto Superiore di Sanità (ISS) manages the antibiotic resistance surveillance project AR-ISS in the human field. Surveillance coordinates a network of hospital microbiology laboratories, which annually provide the ISS with data on antibiotic resistance relating to microbial groups isolated from clinically important infections (bacteremia or meningitis). For each type of microorganism, the focus is mainly on certain antibiotics and, specifically, on certain classes of antibiotics that are particularly important in treatment. The national surveillance of antibiotic resistance in 2019 indicated that in Italy the percentages of resistance to the main classes of antibiotics for the eight pathogens under surveillance (*Staphylococcus aureus, Streptococcus pneumoniae, Enterococcus faecalis, Enterococcus faecium, Escherichia coli, Klebsiella pneumoniae, Pseudomonas aeruginosa* and *Acinetobacter species*) remain high and sometimes increase compared to previous years [6]. *Escherichia coli* are the common enteric bacteria found in wastewater and considered as major indicator of microbial pathogen along with *Enterococci* [7,8,9]. Moreover, as often happens in many viral infections, the most severe patients are at greater risk of developing secondary infections with even fatal consequences [10,11]. As a dramatic and actual example, the coronavirus disease 19 (COVID-19) pandemic spread rapidly in the first half of 2020, absorbing important public health resources, with an impact on health services and direct and indirect consequences on the health of citizens [12,13,14]. Although the World Health Organization (WHO) discourages the use of antibiotics for mild cases of COVID-19 while recommending it for those severe cases at increased risk of secondary bacterial infections and death, the lack of specific therapies for the treatment for this disease has led to the indiscriminate use of various drugs, including antibiotics [15,16,17,18]. Indeed, a recently published meta-analysis estimated that although only 7% of COVID-19 patients had bacterial infection, 70% of COVID-19 patients were treated with antibiotics [19]. The Italian Medicines Agency (AIFA) has produced a report on the consumption of drugs used in the treatment of COVID-19 patients, highlighting how the antibiotic azithromycin, with an increase in use of +195%, is the second molecule after hydroxychloroquine to have undergone the greater difference in consumption between the pre-and post-COVID-19 phase [20]. The increase in the consumption of azithromycin was justified by some evidence that attributed to this antibiotic the ability to modulate the inflammatory response in patients with severe lung disease. Following these observations, its usefulness in therapy in adult patients with COVID-19 is under investigation [21]. Nevertheless, researchers are worried about the increased use of antibiotics during the COVID-19 pandemic considering it as a serious risk factor for the worsening of the threat of antimicrobial resistance, both in the short and long terms [22]. Quaternary ammonium salts (QASs) are cations of general formula [R_1_, R_2_, R_3_ R_4_]N^+^ existing in nature in the form of salts ([R_1_, R_2_, R_3_ R_4_]N^+^X^−^), which have been recently reviewed for their antibacterial activity [23]. Depending on the nature of the substituents, they show different solubility in water. QASs with long-chain substituents are amphiphilic and can be used as surfactants, thus giving these compounds detergent and bactericidal properties. This class of surfactants includes derivatives characterized by monomeric, dimeric, trimeric and polymeric structures.

Dimeric QASs are often named as gemini QAS or gemini surfactants. They are derivatives of QASs that exhibit a broader biocidal activity spectrum than monomeric QASs. In addition to their excellent biocidal properties, gemini surfactants also exhibit better surface properties than monomeric QASs. Gemini surfactants show better wetting properties along with a biodegradability that is comparable to that of monomeric QASs. The mechanism of the biocidal activity of gemini QASs has not been completely understood. It has been evidenced that gemini QASs having 10–12 carbon chains show the highest biocidal activity against bacteria and microscopic fungi. The same authors demonstrated that counterion is also important, as bromides have a higher biocidal activity than the corresponding chlorides [24]. The mechanism of action of QASs was proposed by Sumitomo et al. [25]. In the last decades, much effort has been devoted to the problem of fighting against biofilm formation by bacteria and fungi [26]. Literature provides evidence clearly indicating that surfactants classified as gemini QAS can be used as active compounds in biocidal preparations against the biofilm created by bacteria and fungi [27]. The activity of QASs has been shown to be related to the length of the alkyl chain and the size of the polar head of the surfactant. The biocidal activity of QASs is different towards Gram-positive and Gram-negative bacteria. A biocide is only active when it can pass through the outer layers of the cell, the structure and composition of which allows it to act as a barrier. In the literature, thiourea derivatives with antibacterial activity tested in vitro against pathogenic strains both Gram-positive (*Staphylococcus aureus, Bacillus subtilis* and *Bacillus cereus*) and Gram-negative (*Escherichia coli, Proteus vulgaris* and *Pseudomonas aeruginosa*) have been described and the results have shown considerable activity and a broad spectrum of action [28,29]. The presence of two thiourea moieties, as in the case of bis derivatives, is thought to have a better antibacterial activity, probably due to the fact that the C = S and NH groups of thiourea are easily protonable in acidic conditions and can react with the carboxyl and phosphate groups of the bacterial surface, thus improving the activity. In this paper, four bis-thiourea derivatives (Table 1) have been studied for their antibacterial activity against Gram-positive and Gram-negative bacteria on different bacterial strains isolated from food and environmental matrices. Compounds **1**–**3** were chosen among our QASs previously studied, with alkyl chains of different lengths [30,31,32]. In addition, some guanidine derivatives had shown interesting antibacterial activity, with a mechanism of action based on the strong electrostatic interactions between their positive charges and the electronegative envelope of bacteria [33,34]. Therefore, we thought to synthesize and test compound **4**, bearing two guanidine moieties. 

## 2. Materials and Methods

### 2.1. Thiourea Derivatives

In this study, the antimicrobial activity of four QASs (Table 1) was studied, at a final concentration of 50 mg/mL. The synthesis of compounds **1–3** was previously described [32].

### 2.2. Chemistry

All chemicals were purchased from Sigma-Aldrich (Milan, Italy) or Alfa Aesar (Milan, Italy) at the highest quality commercially available. Solvents were reagent-grade unless otherwise indicated. Yields refer to purified products and were not optimized. Melting point was determined by a Kofler apparatus. ^1^H NMR spectrum was recorded on a Bruker 300 spectrometer operating at 300 MHz and 75 MHz for ^1^H NMR and ^13^C NMR, respectively, in DMSO-d_6_ solvent. Chemical shifts were expressed as δ (ppm). The purity of the compound was checked by TLC (thin layer chromatography), using ethyl acetate as eluent.

#### Synthesis of Pentane-1,5-diyl-bis-S-Amidinothiourea Dihydrobromide (**4**)

Two grams of amidinothiourea (1 equiv.) and 1,5-dibromopentane (0.5 equiv.) were refluxed in ethanol 95% for 18 h. The reaction mixture was evaporated to dryness under vacuum. The residue was washed three times with diethyl ether and then taken up with hot CH_3_CN and crystallized giving **4** (67% yield) as a white solid: Mp 176–177 °C (CH_3_CN); ^1^H NMR (300 MHz, DMSO-d_6_): δ 7.91–6.89 (br s, 12H, NH + NH_2_ + NH_3_^+^); 2.90–2.80 (m, 4H, SCH_2_); 1.54–1.30 (m, 6H, CH_2_); ^13^CNMR (75 MHz, DMSO-d_6_): δ 165.0 (C = NH), 162.4 (C-NH_3_^+^), 30.3 (SCH_2_), 28.4 (CH_2_), 28.3 (CH_2_), 27.9 (CH_2_).

### 2.3. Bacterial Strains and Growth Conditions

The different compounds were tested against a panel of bacterial strains shown in Table 2. A total of thirty-six strains of the culture collection of the Department of Sciences, University of Basilicata, Potenza, Italy, were employed as screening microorganisms for this study. All strains were maintained as freeze-dried stocks in reconstituted (11% *w*/*v*) skim milk, containing 0.1% *w*/*v* ascorbic acid and routinely cultivated in optimal growth conditions [35] (Table 2). These bacteria were chosen to represent the diversity of species of food-borne (labels 1–22; 31–35) and environment-borne (labels 23–30, 36) Gram-positive and Gram-negative bacteria.

### 2.4. Agar Well Diffusion Assay and Minimum Inhibitory Concentration (MIC)

Antimicrobial activity was determined by standard agar well diffusion assay as described by Bonomo et al. [36]. For each strain, a subculture in a specific broth was obtained from the active stock culture by 1% (*v*/*v*) inoculum and incubated overnight at the corresponding culture temperature. 200 μL of each subculture was used to inoculate the agar media (to achieve a final concentration of 10^6^ CFU/mL) and distributed into Petri plates. A volume of 50 μL of each extract was poured into wells (6 mm diameter) bored in the agar plates, and then, the plates were incubated at optimal growth conditions for each strain. Organic solvent was used as negative control while chloramphenicol antibiotic was used as positive control. The experiment was performed in triplicate, and the antimicrobial activity of each extract was expressed in terms of zone of inhibition diameter mean (in mm) produced by the respective extract after 24 h of incubation. An inhibition zone < 10 mm indicated a low antimicrobial activity; 10 < zone of inhibition < 15 mm, a middle antimicrobial activity; a zone of inhibition > 15 mm, a high antimicrobial activity. Then, each compound was screened to determine the MICs in order to evaluate the antimicrobial effectiveness of each compound against different bacterial strains by the agar well diffusion method [37]. Each specific medium inoculated with the strain subculture was distributed into Petri plates, and different concentrations of compounds, ranging from 1.562 mg/mL to 50 mg/mL, were poured into wells bored in the agar plates and the plates were incubated for 24 h. After incubation, the MIC was calculated as the lowest concentration of the compound inhibiting the growth of bacterial strains. The MIC values were done in triplicate.

## 3. Results and Discussion

### 3.1. Chemistry

**Scheme 1:**
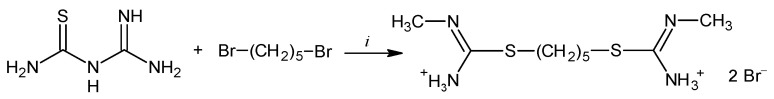
Reagents and conditions: (i) EtOH 95%; RT; 18 h.

Compound **4** was easily prepared as depicted in Scheme 1.

### 3.2. Antimicrobial In Vitro Evaluation

The antimicrobial activity and the MIC of compounds **1–4** were evaluated against selected bacterial strains of significant importance for human health by using the Agar Well Diffusion Assay. Thirty-six Gram-positive and Gram-negative bacteria were used to determine the antimicrobial effect, the action spectrum and the antimicrobial effectiveness of the thiourea derivatives tested. Results showed that compounds demonstrated antimicrobial activity against many tested bacterial species, providing a different inhibitory effect linked to different chemical structure. Some tested bacteria are foodborne; the most of them comes from meat/naturally fermented meat products and the others from milk/milk products; of these, some strains are food spoilage bacteria, and other strains are selected autochthonous starter cultures. The other tested bacterial strains come from an environmental matrix; some are destructive, while others are beneficial and responsible of different processes.

**Figure 1:**
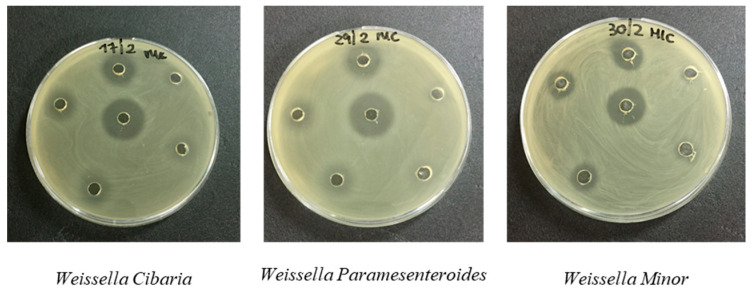
MIC of compound **1** against *Weissella* species.

The results obtained in terms of the diameter of the inhibition zone (expressed in cm) and MIC are reported in Table 3. The thiourea derivative **1** exhibited a broad spectrum of action, showing an inhibitory action against 88.89% of the bacterial strains tested, with an inhibition zone between 1.1 and 3.8 cm. It also showed medium inhibitory activity, with a zone of inhibition between 1.1 and 1.5 cm towards fourteen microorganisms, including the pathogenic strains. A higher inhibitory activity, with an inhibition diameter equal to or greater than 1.6 cm, was found against 47.22% of bacteria, including foodborne species belonging to the genera *Enterococcus*, *Pseudomonas* and *Weissella*, and those of origin of the environmental genus *Bacillus*. Particularly, Gram-positive *Lysinibacillus fusiformis, Bacillus subtilis, Planococcus psychrotoleratus, Bacillus amyloliquefaciens* and *Bacillus cereus* and Gram-negative *Pseudomonas orientalis* bacteria were inhibited at a low concentration (3.125 mg/mL) after treatment with compound **1**; *Weissella confusa* and *Weissella minor* were inhibited at a concentration of 6.25 mg/mL; *Enterococcus caseliflavus, Weissella cibaria, Lactobacillus sakei, Weissella paramesenteroides, Bacillus anthracis* and *Bacillus cereus* showed sensitivity to compound **1** at a concentration of 12.5 mg/mL. The remaining strains required a higher inhibition concentration of 25 mg/mL for approximately 19.44% of the strains and a concentration of 50 mg/mL for 27.78%. Figure 1 shows the inhibition zones of compound **1** against different *Weissella* bacteria.

Compound **2** showed an inhibitory action against 25% of the bacterial strains tested, with an inhibition zone between 1.0 and 2.8 cm. Despite the large inhibition zone found against several Gram-positive and Gram-negative bacteria tested, they showed MIC values of 50 mg/mL. Compound **3** was tested against bacterial strains of environmental matrix and showed an average inhibitory activity, with an inhibition zone between 1.2 and 1.5 cm towards two strains belonging to the genus Bacillus, *Bacillus anthracis* and *Bacillus cereus*. Higher inhibitory activity, indicated by an inhibition diameter greater than 1.6 cm, was found in 66.66% of bacteria tested such as *Lysinibacillus fusiformis, Bacillus subtilis, Planococcus psychrotoleratus, Bacillus amyloliquefaciens, Bacillus cereus* and *Pseudomonas orientalis*. Gram-positive *Lysinibacillus fusiformis* and *Bacillus cereus Bc2* and Gram-negative *Pseudomonas orientalis* species were inhibited at a low concentration (6.25 mg/mL); *Bacillus subtilis, Bacillus amyloliquefaciens* and *Planococcus psychrotoleratus* species were inhibited at a concentration of 12.5 mg/mL. Finally, compound **4** was tested against bacterial strains of food matrix and showed antibacterial activity against 85.19% of species, with an inhibition zone between 0.9 and 2.8 cm, while on the remaining 14.81%, it had no inhibitory activity. This compound showed a high inhibitory activity, evidenced by the formation of an inhibition zone greater than 1.6 cm, against various species, including those belonging to the genera of Gram-positive *Enterococcus* and *Listeria monocytogenes* and Gram-negative *Pseudomonas* and *Escherichia coli*. All strains were inhibited at a very high concentration, with the only exception of Gram-positive *Weissella minor,* which was inhibited at a concentration of 6.25 mg/mL and *Staphylococcus succinus,* which required a MIC of 3.125 mg/mL. The results observed in this study underline the role of the thiourea derivatives in biological activity. As already known in the literature, quaternary ammonium salts have a wide spectrum of biological activity, including the bacteriostatic effect [27]. Compound **2** is a methyl-thiourea with a five-membered alkyl chain (n = 5) while **1** is the corresponding methyl-thiourea with a nine-membered alkyl chain (n = 9). As already reported by Birnie et al. [38], the presence of long alkyl chains promotes the biological activity of thiourea derivatives by increasing lipophilicity and the ability of the compounds to destroy the cell wall of microorganisms. Indeed, compound **1**, bearing the longest alkyl chain of the series, represents the compound with the broadest spectrum of action compared to the other methyl-thioureas. Compound **4**, which bears two guanidine functions, despite the short alkyl chain (n = 5), was found to be active against several Gram-positive bacteria studied (*Staphylococcus* *succinus, Weissella minor,* *Enterococcus casseliflavus, Lactobacillus sakei,* *Listeria monocytogenes*). Compounds **1** and **4** may represent promising tools for future studies in this area.

## 4. Conclusions

In this paper, four bis-thiourea ammonium salts were studied for their antimicrobial activity against different bacterial strains of food and environmental matrices. Compounds with alkyl chains containing odd and even carbon atoms were examined. Among the methyl-thioureas examined, compound **1**, bearing a nine-membered alkyl chain (*n* = 9), was the molecule with the broadest spectrum of action. Compounds **2** and **3**, inferior homologues of compound **1**, with alkyl chains of five and eight carbon atoms, respectively, were less active, confirming that longer alkyl chains may be responsible of the higher biological activity. Compound **4**, a bis-thiourea ammonium salt with two guanidine functions, i.e., the guanidine analogue of **2**, despite the shorter alkyl chain (*n* = 5), showed antibacterial activity against several Gram-positive bacteria, presumably due to the presence of the guanidine moiety. The study herein described may be used as a starting point for further antibacterial studies against Gram-positive and Gram-negative pathogenic and non-pathogenic bacteria of food and environmental matrices. This area is currently receiving particular attention in medicinal chemistry. Future studies to better investigate the exact mechanism of action of gemini QASs, paying special attention to their potential activity on the cell membrane or, even better, on the biofilm formation, would be advisable.

## Figures and Tables

**Table 1:** Thiourea derivatives tested in this study.

Compound	Molecule Structure	Alkyl Chain(CH_2_)_n_
**1**	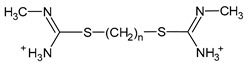	*n* = 9
**2**	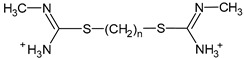	*n* = 5
**3**	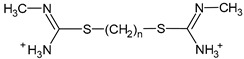	*n* = 8
**4**	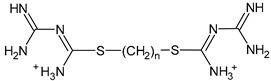	*n* = 5

**Table 2:** Bacterial strains and growth conditions used in this study.

Label	Strain	Bacterial Species	Growth Conditions	
			Temperature	Coltural Medium ^a^
**Gram-positive bacteria**				
*1*	9P	*Carnobacterium maltaromaticum*	20 °C	TSYE Medium
*2*	H02	*Carnobacterium divergens*	20 °C	TSYE Medium
*3*	7R1	*Brochothrix thermosphacta*	20 °C	TSYE Medium
*4*	LMG6399	*Enterococcus hirae*	37 °C	M17 Medium
*5*	ATCC14434	*Enterococcus faecium*	37 °C	M17 Medium
*6*	ATCC14433	*Enterococcus faecalis*	37 °C	M17 Medium
*7*	ATCC14436	*Enterococcus caseliflavus*	37 °C	M17 Medium
*8*	ATCC11576	*Enterococcus durans*	37 °C	M17 Medium
*9*	LMG13129	*Enterococcus gallinarum*	37 °C	M17 Medium
*10*	DSM 20410	*Weissella viridescens*	30 °C	MRS Medium
*11*	DSM 20196	*Weissella confusa*	30 °C	MRS Medium
*12*	DSM 7378	*Weissella hellenica*	30 °C	MRS Medium
*13*	DSM 15878	*Weissella cibaria*	30 °C	MRS Medium
*14*	DBPZ0062	*Lactobacillus sakei Ls1*	30 °C	MRS Medium
*15*	DBPZ0098	*Lactobacillus sakei Ls2*	30 °C	MRS Medium
*16*	DBPZ0224	*Staphylococcus xylosus*	30 °C	TSYE Medium
*17*	DBPZ0251	*Staphylococcus succinus*	30 °C	TSYE Medium
*18*	DBPZ0241	*Staphylococcus equorum*	30 °C	TSYE Medium
*19*	BL/26	*Listeria innocua*	30 °C	TSYE Medium
*20*	DSM20288	*Weissella paramesenteroides*	30 °C	MRS Medium
*21*	DSM20014	*Weissella minor*	30 °C	MRS Medium
*22*	DBPZ001	*Listeria monocytogenes*	30 °C	TSYE Medium
*23*	DSPZA11II	*Lysinibacillus fusiformis*	30 °C	Plate count agar
*24*	DSPZA12TII	*Bacillus subtilis*	30 °C	Plate count agar
*25*	DSPZA2TI	*Planococcus psychrotoleratus*	30 °C	Plate count agar
*26*	DSPZA5TI	*Bacillus amyloliquefaciens*	30 °C	Plate count agar
*27*	DSPZA8TII	*Bacillus anthracis*	30 °C	Plate count agar
*28*	DSPZA102II	*Bacillus amyloliquefaciens*	30 °C	Plate count agar
*29*	DSPZA4I	*Bacillus cereus Bc1*	30 °C	Plate count agar
*30*	DSPZA191I	*Bacillus cereus Bc2*	30 °C	Plate count agar
**Gram-negative bacteria**				
*31*	6P2	*Pseudomonas fragi*	20 °C	TSYE Medium
*32*	53M	*Hafnia alvei*	30 °C	TSYE Medium
*33*	42M	*Pseudomonas proteamaculans*	30 °C	TSYE Medium
*34*	32	*Escherichia coli*	37 °C	TSYE Medium
*35*	DBPZ002	*Salmonella serovar*	30 °C	TSYE Medium
*36*	DSPZA141II	*Pseudomonas orientalis*	30 °C	Plate count agar

^a^ TSYE = Tryptone Soya Yeast Extract.

**Table 3:** Antibacterial activity of compounds **1**–**4**.

		Compound 1		Compound 2		Compound 3		Compound 4	
Label	Bacterial Strains	Inhibition Zone ^a^	MIC (mg/mL)	Inhibition Zone ^a^	MIC (mg/mL)	Inhibition Zone ^a^	MIC (mg/mL)	Inhibition Zone ^a^	MIC (mg/mL)
**Gram-positive bacteria**									
*1*	*Carnobacterium maltaromaticum*	1.1 ± 0.21	50 ± 0.67	/	/	/	/	/	/
*2*	*Carnobacterium divergens*	/	/	/	/	/	/	/	/
*3*	*Brochothrix thermosphacta*	/	/	/	/	/	/	/	/
*4*	*Enterococcus hirae*	1.5 ± 0.34	25 ± 0.81	/	/	/	/	1.3 ± 0.76	25 ± 0.69
*5*	*Enterococcus faecium*	1.5 ± 0.11	50 ± 0.99	/	/	/	/	1.4 ± 0.48	50 ± 0.36
*6*	*Enterococcus faecalis*	1.6 ± 0.07	25 ± 0.21	/	/	/	/	1.5 ± 0.05	50 ± 0.08
*7*	*Enterococcus casseliflavus*	2.0 ± 0.08	12.5 ± 0.66	/	/	/	/	1.9 ± 0.08	12.5 ± 0.22
*8*	*Enterococcus durans*	2.0 ± 0.11	50 ± 0.88	/	/	/	/	1.7 ± 0.77	50 ± 0.88
*9*	*Enterococcus gallinarum*	1.5 ± 0.03	25 ± 0.79	/	/	/	/	2.0 ± 0.34	12.5 ± 0.90
*10*	*Weissella viridescens*	/	/	1.2 ± 0.08	50 ± 0.90	/	/	/	/
*11*	*Weissella confusa*	2.0 ± 0.65	6.25 ± 0.78	1.4 ± 0.15	50 ± 0.22	/	/	1.6 ± 0.66	50 ± 0.05
*12*	*Weissella hellenica*	1.4 ± 0.37	25 ± 0.67	/	/	/	/	1.2 ± 0.80	25 ± 0.78
*13*	*Weissella cibaria*	2.0 ± 0.12	12.5 ± 0.79	/	/	/	/	1.5 ± 0.37	50 ± 0.88
*14*	*Lactobacillus sakei Ls1*	1.4 ± 0.69	12.5 ± 0.62	/	/	/	/	1.6 ± 0.79	12.5 ± 0.99
*15*	*Lactobacillus sakei Ls2*	1.6 ± 0.67	12.5 ± 0.04	/	/	/	/	1.5 ± 0.04	12.5 ± 0.08
*16*	*Staphylococcus xylosus*	1.5 ± 0.70	50 ± 0.09	/	/	/	/	1.7 ± 0.72	50 ± 0.36
*17*	*Staphylococcus succinus*	1.7 ± 0.11	50 ± 0.55	1.2 ± 0.92	50 ± 0.77	/	/	2.8 ± 0.06	3.125 ± 0.54
*18*	*Staphylococcus equorum*	1.3 ± 0.38	25 ± 0.86	/	/	/	/	0.9 ± 0.09	50 ± 0.89
*19*	*Listeria innocua*	1.2 ± 0.13	50 ± 0.02	/	/	/	/	1.0 ± 0.15	50 ± 0.32
*20*	*Weissella paramesenteroides*	2.0 ± 0.10	12.5 ± 0.08	/	/	/	/	1.0 ± 0.78	50 ± 0.21
*21*	*Weissella minor*	1.6 ± 0.06	6.25 ± 0.91	/	/	/	/	1.5 ± 0.92	6.25 ± 0.61
*22*	*Listeria monocytogenes*	1.5 ± 0.17	25 ± 0.07	/	/	/	/	1.6 ± 0.55	12.5 ± 0.45
*23*	*Lysinibacillus fusiformis*	3.1 ± 0.09	3.125 ± 0.65	2.8 ± 0.87	50 ± 0.34	2.3 ± 0.08	6.25 ± 0.66	/	/
*24*	*Bacillus subtilis*	3.2 ± 0.11	3.125 ± 0.15	1.7 ± 0.90	50 ± 0.08	1.7 ± 0.32	12.5 ± 0.08	/	/
*25*	*Planococcus psychrotoleratus*	1.3 ± 0.55	3.125 ± 0.03	/	/	2.1 ± 0.97	12.5 ± 0.70	/	/
*26*	*Bacillus amyloliquefaciens*	3.8 ± 0.72	3.125 ± 0.42	/	/	3.0 ± 0.77	12.5 ± 0.22	/	/
*27*	*Bacillus anthracis*	1.5 ± 0.04	12.5 ± 0.90	/	/	1.5 ± 0.58	25 ± 0.77	/	/
*28*	*Bacillus amyloliquefaciens*	1.5 ± 0.18	50 ± 0.65	/	/	/	/	/	/
*29*	*Bacillus cereus Bc1*	2.0 ± 0.78	12.5 ± 0.43	/	/	1.2 ± 0.72	50 ± 0.09	/	/
*30*	*Bacillus cereus Bc2*	2.6 ± 0.44	3.125 ± 0.75	2.4 ± 0.88	50 ± 0.77	2.7 ± 0.98	6.25 ± 0.54	/	/
**Gram-negative bacteria**									
*31*	*Pseudomonas fragi*	2.0 ± 0.43	50 ± 0.28	1.3 ± 0.77	50 ± 0.95	/	/	1.9 ± 0.98	50 ± 0.09
*32*	*Hafnia alvei*	/	/	/	/	/	/	1.3 ± 0.44	50 ± 0.88
*33*	*Pseudomonas proteamaculans*	1.4 ± 0.76	25 ± 0.98	/	/	/	/	1.6 ± 0.90	50 ± 0.67
*34*	*Escherichia coli*	2.0 ± 0.90	50 ± 0.75	/	/	/	/	2.5 ± 0.70	50 ± 0.07
*35*	*Salmonella serovar*	1.5 ± 0.35	50 ± 0.91	1.1 ± 0.34	50 ± 0.36	/	/	1.0 ± 0.39	50 ± 0.77
*36*	*Pseudomonas orientalis*	3.3 ± 0.22	3.125 ± 0.23	1.0 ± 0.58	50 ± 0.87	3.2 ± 0.40	6.25 ± 0.38	/	/

^a^ diameter in cm.

## Data Availability

The data presented in this study are available in manuscript.

## Abbreviations

AIFA, Italian Medicines Agency; AMR, antimicrobial resistance; AR-ISS, antibiotic resistance surveillance project; CLSI, Clinical Laboratory Standards Institute; COVID-19, Coronavirus Disease 19; ISS, Istituto Superiore di Sanità; MICs, Minimum Inhibitory Concentrations; PNCAR, National Action Plan on Antimicrobial Resistance; QASs, quaternary ammonium salts; WHO, World Health Organization.

## References

[1] Ferri M., Ranucci E., Romagnoli P., Giaccone V. (2017). Antimicrobial resistance: A global emerging threat to public health systems. Crit. Rev. Food Sci. Nutr..

[2] De Rosa M., Bonomo M.G., Vassallo A., Palma G., Calabrone L., Bimonte S., Silvestris N., Amruthraj N.J., Sinicropi M.S., Salzano G. (2018). Linezolid and its derivatives: The promising therapeutic challenge to multidrug-resistant pathogens. Pharmacologyonline.

[3] Bonomo M.G., Calabrone L., Saturnino C., Sinicropi M.S., Capasso A., Salzano G. (2019). Antibacterial activity of new β-lactam compound. PharmacologyOnLine.

[4] De Kraker M.E.A., Stewardson A.J., Harbarth S. (2016). Will 10 million people die a year due to antimicrobial resistance by 2050?. PLoS Med..

[5] Barchitta M., Quattrocchi A., Maugeri A., La Rosa M.C., La Mastra C., Sessa L., Cananzi P., Murolo G., Oteri A., Basile G. (2019). Antibiotic consumption and resistance during a 3-year period in Sicily, Southern Italy. Int. J. Environ. Res. Public Health.

[6] Pantaleo E., Gamalero E., Leli C., Rocchetti A. (2021). Il sistema di sorveglianza dell’antibiotico resistenza AR-ISS: Uno strumento efficace per migliorare la gestione degli antibiotici. Work. Pap. Public Health.

[7] Santoro A., Cappello A.R., Madeo M., Martello E., Iacopetta D., Dolce V. (2011). Interaction of fosfomycin with the glycerol 3-phosphate transporter of *Escherichia coli*. Biochim. Biophys. Acta.

[8] Pozzi C., Ferrari S., Cortesi D., Luciani R., Stroud R.M., Catalano A., Costi M.P., Mangani S. (2012). The structure of *Enterococcus faecalis* thymidylate synthase provides clues about folate bacterial metabolism. Acta Crystallogr. D Biol. Crystallogr..

[9] Bonomo M.G., Salzano G. (2012). Microbial diversity and dynamics of Pecorino di Filiano PDO, a traditional cheese of Basilicata region (Southern Italy). Int. J. Dairy Tech.

[10] Zhou F., Yu T., Du R., Fan G., Liu Y., Liu Z., Xiang J., Wang Y., Song B., Gu X. (2020). Clinical course and risk factors for mortality of adult in patients with COVID-19 in Wuhan, China: A retrospective cohort study. Lancet.

[11] Caruso A., Ceramella J., Iacopetta D., Saturnino C., Mauro M.V., Bruno R., Aquaro S., Sinicropi M.S. (2019). Carbazole derivatives as antiviral agents: An overview. Molecules.

[12] Driggin E., Madhavan M.V., Bikdeli B., Chuich T., Laracy J., Bondi-Zoccai G., Brown T.S., Nigoghossian C.D., Zidar D.A., Haythe J. (2020). Cardiovascular considerations for patients, health care workers, and health systems during the Coronavirus Disease 2019 (COVID-19) Pandemic. J. Am. Coll. Cardiol..

[13] Giuzio F., Bonomo M.G., Armenante G., Barra G., Casolaro G., Di Ludovico C., Galasso L., Giacco D., Grassi F., Lardo D. (2021). The monitoring model for covid-19 patients in the context of territorial medicine: The experience of the covid special unit (usco) of potenza. Pharmacologyonline.

[14] Catalano A. (2020). COVID-19: Could irisin become the handyman myokine of the 21st century?. Coronaviruses.

[15] Catalano A., Iacopetta D., Pellegrino M., Aquaro S., Franchini C., Sinicropi M. (2021). Diarylureas: Repositioning from antitumor to antimicrobials or multi-target agents against new pandemics. Antibiotics.

[16] Saturnino C., Grande F., Aquaro S., Caruso A., Iacopetta D., Bonomo M.G., Longo P., Schols D., Sinicropi M.S. (2018). Chloro-1,4-dimethyl-9*H*-carbazole derivatives displaying anti-HIV activity. Molecules.

[17] Tabbi A., Tebbani D., Caporale A., Saturnino C., Nabavi S.M., Palma G., Arra C., Cantürk Z., Turan-Zitouni G., Merazig H. (2017). New adamantyl chalcones: Synthesis, antimicrobial and anticancer activities. Curr Top. Med Chem.

[18] Bonomo M.G., Milella L., Martelli G., Salzano G. (2013). Stress response assessment of *Lactobacillus sakei* strains selected as potential autochthonous starter cultures by flow cytometry and nucleic acid double staining analyses. J. Appl. Microbiol..

[19] Langford B.J., So M., Raybardhan S., Leung V., Westwood D., MacFadden D.R., Soucy J.R., Daneman N. (2020). Bacterial co-infection and secondary infection in patients with COVID-19: A living rapid review and meta-analysis. Clin. Microbiol. Infect..

[20] Lagolio E., Valbonesi S. (2020). L’antibiotico-resistenza al tempo del COVID-19: Tattiche e strategie. Med. Prat..

[21] Chilamakuri R., Agarwal S. (2021). COVID-19: Characteristics and Therapeutics. Cells.

[22] Hsu J. (2020). How COVID-19 is accelerating the threat of antimicrobial resistance. BMJ.

[23] Francomano F., Caruso A., Barbarossa A., Fazio A., La Torre C., Ceramella J., Mallamaci R., Saturnino C., Iacopetta D., Sinicropi M.S. (2019). β-caryophyllene: A sesquiterpene with countless biological properties. Appl. Sci..

[24] Koziróg A., Kregiel D., Brycki B. (2017). Action of monomeric/gemini surfactants on free cells and biofilm of Asaia lannensis. Molecules.

[25] Sumitomo T., Maeda T., Nagamune H., Kourai H. (2004). Bacterioclastic action of a bis-quaternary ammonium compound against *Escherichia coli*. Biocontrol Sci..

[26] Rosato A., Catalano A., Carocci A., Carrieri A., Carone A., Caggiano G., Franchini C., Corbo F., Montagna M.T. (2016). In vitro interactions between anidulafungin and nonsteroidal anti-inflammatory drugs on biofilms of *Candida* spp.. Bioorg. Med. Chem..

[27] Kwaśniewska D., Chen Y.-L., Wieczorek D. (2020). Biological activity of quaternary ammonium salts and their derivatives. Pathogens.

[28] Hashem H.E., Amr A.E.G.E., Nossier E.S., Elsayed E.A., Azmy E.M. (2020). Synthesis, antimicrobial activity and molecular docking of novel thiourea derivatives tagged with thiadiazole, imidazole and triazine moieties as potential DNA gyrase and topoisomerase IV inhibitors. Molecules.

[29] De Rosa M., Zanfardino A., Notomista E., Wichelhaus T.A., Saturnino C., Varcamonti M., Soriente A. (2013). Novel promising linezolid analogues: Rational design, synthesis and biological evaluation. Eur. J. Med. Chem..

[30] Ceramella J., Mariconda A., Rosano C., Iacopetta D., Caruso A., Longo P., Sinicropi M.S., Saturnino C. (2020). α–ω Alkenyl-bis-S-guanidine thiourea dihydrobromide affects HeLa cell growth hampering tubulin polymerization. ChemMedChem.

[31] Paesano N., Marzocco S., Vicidomini C., Saturnino C., Autore G., De Martino G., Sbardella G. (2005). Synthesis and biological evaluation of 3-benzyl-1-methyl- and 1-methyl-3-phenyl-isothioureas as potential inhibitors of iNOS. Bioorg. Med. Chem. Lett..

[32] Saturnino C., Buonerba M., Paesano N., Lancelot J.C., De Martino G. (2003). In vitro anti-acanthamoeba action by thioureidic derivatives. Il Farm..

[33] Pasero C., D’Agostino I., De Luca F., Zamperini C., Deodato D., Truglio G.I., Sannio F., Del Prete R., Ferraro T., Visaggio D. (2018). Alkyl-guanidine compounds as potent broad-spectrum antibacterial agents: Chemical library extension and biological characterization. J. Med. Chem..

[34] Zamperini C., Maccari G., Deodato D., Pasero C., D’Agostino I., Orofino F., De Luca F., Dreassi E., Docquier J.D., Botta M. (2017). Identification, synthesis and biological activity of alkyl-guanidine oligomers as potent antibacterial agents. Scient. Rep..

[35] Bonomo M.G., Cafaro C., Russo D., Faraone I., Milella L., Saturnino C., Capasso A., Sinicropi M.S., Salzano G. (2020). Antimicrobial and antioxidant properties and phytochemical screening of different mother tinctures against food-borne bacteria. Pharmacologyonline.

[36] Bonomo M.G., Cafaro C., Russo D., Calabrone L., Milella L., Saturnino C., Capasso A., Salzano G. (2020). Antimicrobial activity, antioxidant properties and phytochemical screening of *Aesculus hippocastanum* mother tincture against food-borne bacteria. Lett. Drug Des. Discov..

[37] CLSI (2012). Methods for Dilution Antimicrobial Susceptibility Tests for Bacteria that Grow Aerobically, Approved [Document M7-A9].

[38] Birnie C.R., Malamud D., Schnaare R.L. (2000). Antimicrobial evaluation of *N*-alkyl betaines and *N*-alkyl-*N, N*-dimethylamine oxides with variations in chain length. Antimicrob. Agents Chemother..

